# Painful, Tender, Localized, Idiopathic Livedo Reticularis

**DOI:** 10.7759/cureus.52311

**Published:** 2024-01-15

**Authors:** Yanci A Algarin, Robert Pariser

**Affiliations:** 1 Dermatology, Eastern Virginia Medical School, Norfolk, USA

**Keywords:** skin, biopsy, idiopathic, tender, livedo reticularis

## Abstract

Livedo reticularis (LR) is a unique cutaneous condition characterized by a reddish-blue to purple, net-like cyanosis of the skin, often associated with disturbances in cutaneous blood flow. This case report discusses a 30-year-old woman with a history of Hashimoto thyroiditis, vitamin D deficiency, migraines, and goiter who presents with painful, localized LR on her right flank. Despite her extensive medical history, there were no significant findings in her laboratory and imaging studies, including a normal epidermis in skin biopsies. The LR in this case is distinguished by its persistence and the presence of pain, a symptom not commonly associated with LR. Various treatments, including 5% lidocaine ointment, oral analgesics, and gabapentin, were considered, but her symptoms remained stable over 13 months. This case exemplifies the complexity of LR, particularly when presenting with atypical symptoms like pain. It highlights the need for further research into the pathophysiology and treatment of LR, especially in cases deviating from the typical symptomatology, and suggests the potential value of a multi-disciplinary approach to management.

## Introduction

Livedo reticularis (LR) is a distinctive cutaneous condition described by Hebra more than a century ago [1]. LR manifests as a transient or persistent pattern of reddish-blue to purple, net-like cyanosis on the skin's surface. This vascular pattern is a consequence of disruptions in the flow of blood through the skin, which can occur in various physiologic and pathologic states. While LR can manifest idiopathically (physiological LR) or from vascular disease (pathologic LR), tenderness or pain are generally absent [1-2]. We report a case of LR in a 30-year-old woman with persistent, localized LR, for which the chief complaint was pain and tenderness.

## Case presentation

**Figure 1:**
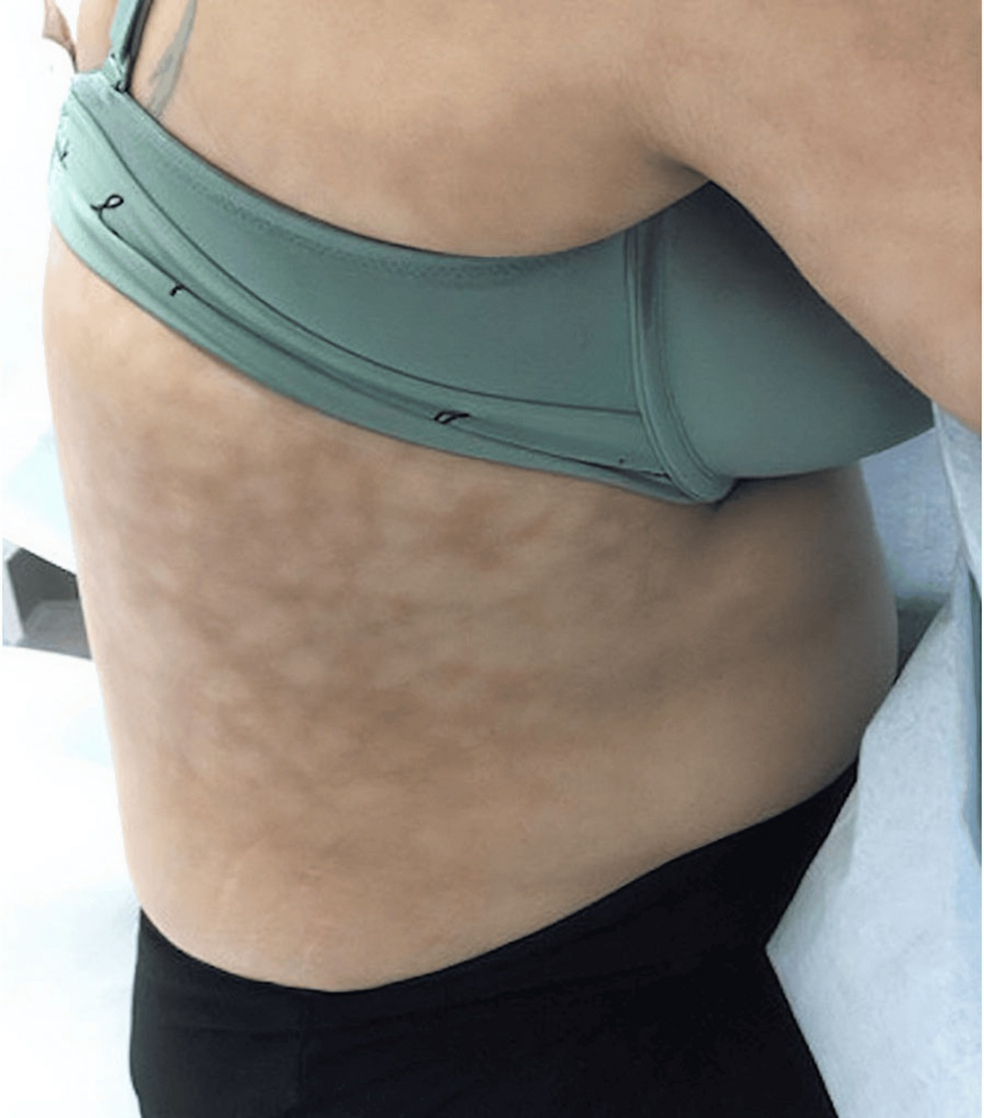
Clinical image of the right flank Network-like, tender, painful, purplish vascular pattern localized to the right flank

**Figure 2:**
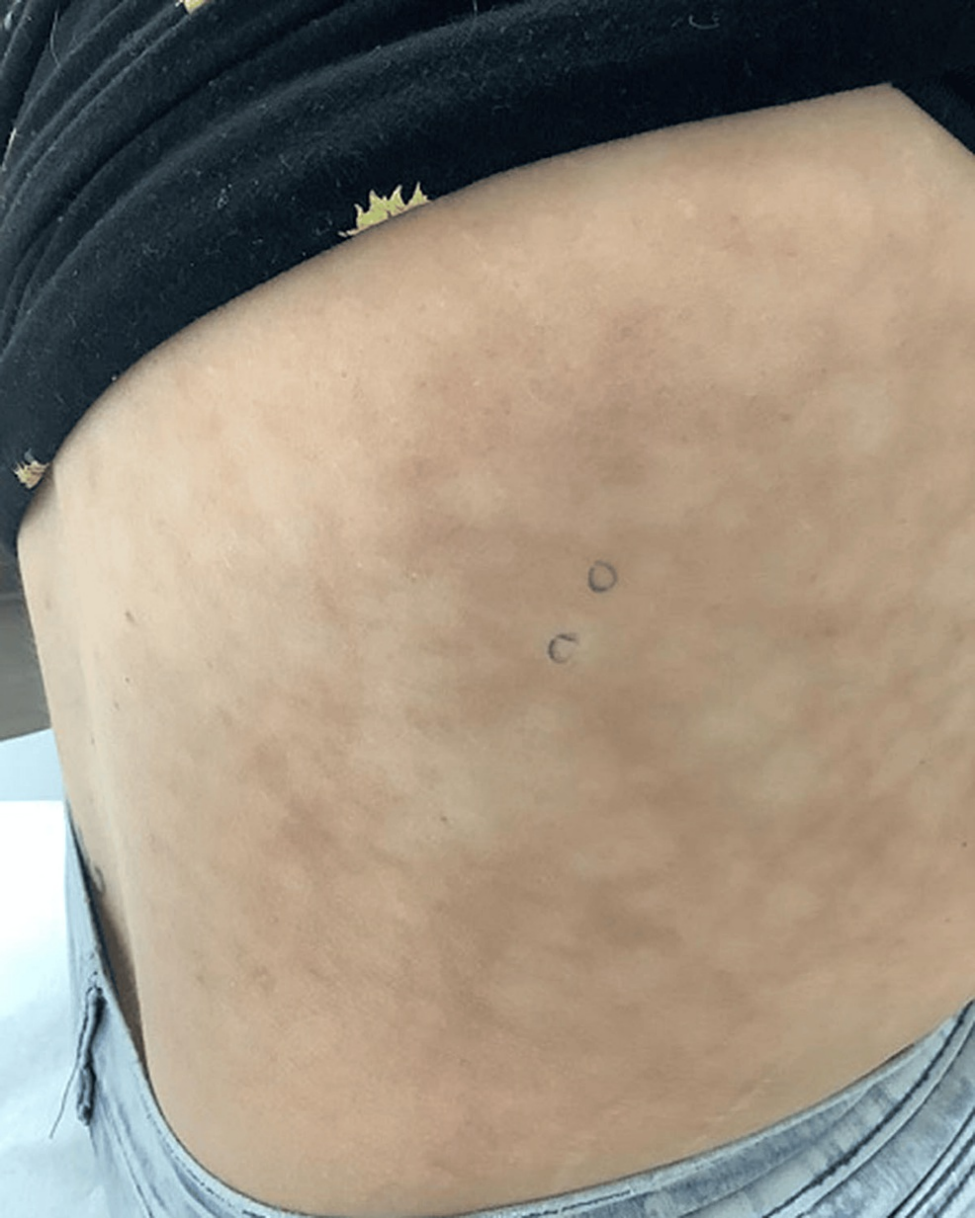
Clinical image of the right flank Biopsy sites of both involved (purplish vascular pattern) and uninvolved skin

A 30-year-old woman presented with a purplish-tan, blanchable, network-like vascular pattern on the right flank (Figures 1-2). Oral and genital mucosae, palms and soles, abdomen, trunk, scalp, and nails were normal. Her medical history included Hashimoto thyroiditis, vitamin D deficiency, migraines, and goiter. She denied surgery or trauma in the affected area, nor were there any systemic symptoms such as fever, emesis, chronic diarrhea, cough, weight loss, or neck stiffness. There was no history of oral ulcers, photosensitivity, joint pain, muscle weakness, skin thickening, or Raynaud's phenomenon. Medications included tramadol, amitriptyline, galcanezumab, levothyroxine, magnesium oxide, metformin, prednisone, spironolactone, and topiramate. The patient reported pain in the area, and there was tenderness on palpation. She denied any possibility that the affected area had been subjected to localized heating from any source. The condition had been stable for 10 months as of her last clinic visit. No other abnormal vascular skin lesions were present. Aspirin and tramadol affected neither her symptoms nor the appearance of the lesion. Laboratory studies and imaging were completed prior to her visit with us. These included complete blood count, complete metabolic panel, erythrocyte sedimentation rate, renal function test, lipid profile, fasting and postprandial blood sugar, urinalysis, 25-hydroxy vitamin D test, human immunodeficiency virus enzyme-linked immunosorbent assay test, hepatitis B antigen, anti-hepatitis C virus antibodies, thyroid functions, antinuclear antibody, complement proteins C3 and C4, rheumatoid factor, antineutrophilic cytoplasmic antibody panel, cryoglobulins, and chest X-ray, all of which were unremarkable.

A computed tomography study before and after an intravenous contrast injection showed no vascular anomalies. A punch biopsy was obtained from an area of reddish-tan pigmentation on the right and from a nearby area of normally pigmented skin for comparison. Both specimens showed a normal epidermis, a sparse perivascular infiltrate, and no evidence of thrombosis. At her last clinic visit, the patient was prescribed a treatment regimen that included 5% lidocaine ointment in addition to her ongoing oral analgesics (acetaminophen and ibuprofen). However, based on a follow-up telephone conversation 13 months after the onset of her symptoms, it was confirmed that her symptoms have remained stable without significant improvement despite using these prescribed medications as instructed. Gabapentin was considered, but the patient has not returned for further follow-up.

## Discussion

In 1907, Ehrmann described two distinct manifestations of LR: a physiological type characterized by complete (unbroken) circular patterns, and a pathological type known as livedo racemosa, notable for its incomplete (broken) and disrupted patterns [3]. The visible discoloration in both types arises from diminished circulation and reduced oxygen levels at the outermost regions of the skin [4-5]. Barker et al. consider LR a circulatory phenomenon rather than a disease [6]. LR lacking systemic associations can be categorized into three distinct types: physiologic, primary, and idiopathic [7].

Physiologic LR, or cutis marmorata, typically manifests in response to colder temperatures and is primarily seen in preterm infants, neonates, and fair-skinned individuals. It most commonly affects the lower extremities and resolves completely with a warming of the affected limb [7].

Primary LR is a diagnosis of exclusion. It has a fluctuating course, yet it is distinct from cutis marmorata because the alterations in skin color do not correlate with environmental temperature or systemic factors [7].

Idiopathic LR is persistent and unresolving. Although it has many similarities with primary LR, it is distinguished by the persistence of the livedo pattern [7]. The diagnosis requires the absence of related, underlying pathology.

Secondary LR is associated with a number of underlying conditions, including hematological conditions, autoimmune disorders, infections, neurological issues, and medications such as minocycline, amantadine, and gemcitabine [2,5,7].

Livedoid vasculopathy (LV) is a rare ulcerative subtype of the pathological livedo racemosa caused by fibrinolytic irregularities and microcirculatory thrombosis. Clinical features of LV include recurrent, severely painful ulcers, violaceous skin discoloration, and atrophie blanche [8].

When assessing patients with LR, a thorough examination of their medical history and physical condition is essential. To comprehensively evaluate the potential systemic diseases associated with LR, a range of laboratory investigations prove valuable, including a complete blood count, platelet count, coagulation profile, assessment of cryoproteins, testing for antinuclear antibodies, and screening for anticardiolipin antibodies. These screening tests play a crucial role in identifying the presence of significant systemic diseases linked to LR.

Skin biopsies have proven useful in diagnosing various causes of LR, aiding in the differentiation between vasculitis, vasculopathy, and normal tissue. However, the overall diagnostic yield of these biopsies tends to be relatively low [2]. Consequently, in cases where the cause of LR remains unidentified, especially when a systemic disorder is suspected, it is advisable to conduct multiple punch biopsies. This should include at least one biopsy from a centrally blanched area and another from a peripherally bluish region, as the use of multiple biopsies enhances the likelihood of reaching a definitive diagnosis. If nodules and fixed purpuric areas are present, a biopsy of these areas is recommended. Importantly, the primary objective of a biopsy is to collect tissue from the deeper layers of the skin, particularly from the medium-sized blood vessels in the deep reticular dermis and the fat beneath it. To achieve this, opting for either a wedge biopsy or a larger punch biopsy proves most effective in obtaining valuable diagnostic information [2].

Currently, there is no established optimal treatment for LR. Cold exposure should be avoided in cases of idiopathic LR. Attempts at management have included antiplatelet medications like low-dose aspirin and clopidogrel, as well as vasodilators such as nifedipine and pentoxifylline. Other measures to reduce risk involve controlling blood pressure, managing diabetes, quitting smoking, and weight loss. These therapies are inconsistently effective [9]. Pain secondary to LR is uncommon. As with cases of LV, one can consider nonsteroidal anti-inflammatory medications like indomethacin or acetaminophen to manage discomfort. For neuropathic discomfort, options such as tricyclic antidepressants, gabapentin, pregabalin, or carbamazepine can be considered, particularly for those experiencing chronic pain as seen in cases of LV [8]. If underlying disorders are discovered, the focus of treatment should be on addressing these specific conditions.

## Conclusions

We present a case of persistent, painful, idiopathic, localized, recalcitrant LR in a 30-year-old woman. This case of LR can be reasonably classified as idiopathic, following the exclusion of various potential etiologies. Such classification anticipates the persistence of the lesion; however, the symptomatic manifestation of this process was unanticipated. Despite various investigations and symptomatic management, the condition has remained stable. This case underscores the complexity of LR and its potential to manifest in atypical and discomforting ways. We cannot definitely conclude that the symptomatic nature is a specific characteristic of idiopathic LR, given the scarcity of evidence linking pain with LR. Further research is needed to better understand the pathophysiology of and the treatment options for LR, especially in cases with atypical symptomatic presentations. Furthermore, a multi-disciplinary approach may be necessary for effective and proper treatment.
